# Dexmedetomidine: Current Role in Burn ICU

**Published:** 2017-07-01

**Authors:** G Scibelli, L Maio, M Sasso, A Lanza, G Savoia

**Affiliations:** 1 Anaesthesia and Intensive Care ASL Caserta; 2 Anaesthesia and Intensive Care Department - AORN “A. Cardarelli”-Napoli

**Keywords:** dexmedetomidine, burns, ICU, sepsis

## Abstract

Dexmedetomidine (DEX) is a relativelyrecent a_2_-adrenergic agonist which provides sedation, anxiolysis and analgesia with much less respiratory depression than other sedatives. These characteristics have implemented the use of the drug in the ICUs in order to achieve the target of a “arousable sedation”, thanks to its significant manageability. Its sedative-analgesic properties are also particularly suitable for use in burn ICUs, both adult and pediatric, which is why the current Guidelines have recognized a central role in the management of these categories of patients. Finally, DEX has showed significant anti-inflammatory effect both in animal models and in preliminary clinical trials, reducing vasopressor requirements and main mediators levels of the systemic inflammatory response involved in sepsis and similar processes, suggesting its use for improved outcome in ICU septic patients.

## I. INTRODUCTION

Dexmedetomidine (DEX) is a novel α_2_-adrenergic agonist that provides sedation, anxiolysis and analgesia with much less respiratory depression than other sedatives [1]. It is a selective α_2_-adrenoceptor agonist similar to Clonidine of the imidazole subclass [2].

The mechanism of sedation by α_2_-agonists is thought to occur via activation of presynaptic receptors in the reticular formation, inhibiting the release of norepinephrine, primarily at the locus ceruleus. The analgesic effects and potentiation of opioid-induced analgesia result from the activation of α_2_-adrenergic receptors in the dorsal horn of the spinal cord and inhibition of substance-P release [3]. Thus, these drugs have been noted to reduce the need for narcotics. The end-organ effects are mediated via postsynaptic α_2_-adrenergic receptors and subsequent activation of a pertussis toxin-sensitive guanine nucleotide regulatory protein (G protein) [3].

The bradycardia associated with DEX is caused by activation of receptors in the medullary vasomotor center, reducing norepinephrine with resultant central sympatholytic effect: decreased heart rate and blood pressure [3]. Respiratory drive appears to be maintained during DEX administration.

DEX exhibits a higher specificity for α_2_-receptors and has a shorter half-life than Clonidine (2–3 vs 12–24 hours) [3]. Its elimination half-life is approximately 2 h after a rapid distribution phase of about 6 min, with mainly renal elimination (95%). The drug is metabolized in the liver via N-glucuronidation, N-methylation, and cytochrome P450, then excreted primarily in the urine. There are no active or toxic metabolites. It can be antagonized if needed by administration of atipamezole [4].

Contraindications include hypersensitivity to DEX or any component, as well as use outside of the intensive care setting. Adverse reactions are primarily cardiogenic, with bradycardia and sinus arrest associated with rapid intravenous administration. Hypotension is also a possible adverse effect. There is also concern for withdrawal symptoms similar to Clonidine if used for prolonged periods or abruptly discontinued [5].

DEX produces “arousable sedation” and analgesia with prevention of recall and memory at higher doses [3]. DEX sedation exhibits properties similar to natural sleep, specifically simulating nonrapid eye movement sleep.

The stated uses are:

1. sedation of initially intubated and mechanically ventilated patients during treatment in an intensive care unit (ICU);
2. sedation prior to and/or during surgical or other procedures of nonintubated patients;
3. premedication prior to anesthesia induction;
4. treatment of shivering;
5. premedication to attenuate the cardiostimulatory and postanesthetic delirium of ketamine.

DEX is approved by the FDA for use in people aged ≥18 years [2].

## II. ROLE IN ICU

A large body of recent work supports its favorable profile in improving outcome from ICU delirium [6], for opioid, benzodiazepine, and alcohol withdrawal [7], as well as for sedation during monitored anesthesia care and regional anesthesia [8].

The goal of sedation in the ICU is a patient who is calm, possibly lightly asleep, but easily arousable. Many drugs have been used, including ketamine, inhalational anesthetics, tranquilizers, and benzodiazepines [9,10]. Continuous midazolam infusion is commonly used in ICUs, but often patients develop a tolerance to the drug[11]. This can lead to increasing doses of midazolam as well as inadequate sedation [11,12,13]. Other significant physiological effects at therapeutic doses include a slight reduction in heart rate, systemic vascular resistance, and a small reduction in tidal volume with a compensatory increased respiratory rate. Potential complications thus include hypotension, respiratory depression, and oversedation [13].

A prospective, double-blind, randomized trial in 68 medical centers in five countries found that DEX and midazolam produced equivalent sedation in adult ICU patients. During the study, DEX was used for a shorter time period because of more rapid extubation. Patients treated with DEX were found to have significant decrease in delirium as well. The main complication was bradycardia (42.2% of patients), while 4.9% of patients required an intervention, either titration of DEX or atropine. Alternatively, there was a higher percentage of patients who had tachycardia and hypertension in the midazolam group. There were no episodes of rebound hypertension or tachycardia in the DEX group [14].

Benzodiazepines disrupt the normal electroencephalographic patterns of sleep, and these effects may be responsible for the delirium seen in the ICU setting. Because DEX allows patients to get a more healthy sleep pattern, they may theoretically be able to avoid ICU delirium and the increased complications therein^3^. Each additional day of delirium increases the risk of prolonged hospitalization by 20% and increases the likelihood of a poor functional status at 3 and 6 months [14].

In a recent (2010) meta-analysis on the analgesic and sedative effects of DEX on patients requiring mechanical ventilation in the ICU, Tan and Ho showed that DEX may reduce the length of stay of some ICU patients but had no effect on days of ventilation [15].

An updated meta-analysis of all RCTs on DEX was recently made by Pasin et al. [16] in order to compare the drug to alternative systems used in ICU settings, evaluating some parameters such as time to extubation, ICU stay and survival. 27 manuscripts were admitted to the study, including 3648 (Dex group: 1870; controls: 1778) heterogeneous patients, especially in ICU settings: general ICU (13 trials), cardiac surgery ICU (10 trials), major non-cardiac surgery ICU (4 Trials) and admission to the ICU after cesarean section-eclampsia (1 trial). In 18 trials a loading dose between 0.1 to 6 mcg/kg preceded the continuous infusion, this one the only technique used in 6 trials and ranged between 0.1 to 2.5 mcg/kg/h. In one trial only the loading dose was used and one trial gave no details.

DEX was compared to propofol, midazolam, placebo, morphine, haloperidol and lorazepam.

The main results of this meta-analysis showed that DEX group is associated with a reduction in ICU stay, time of extubation, rescue dose of sedative or analgesic drugs; on the other hand, with an increased risk of bradycardia and hypotension, but without significant worsening of mortality [16].

## III. ROLE IN BURN ICU - PATHOPHYSIOLOGY OF PAIN IN BURN PATIENTS

The pain is a fundamental aspect in the management of burn patients [17]. In fact, the prolonged exposure to painful stimuli, directly related to the burned surface, causes centralization of pain and persistent pain [18,19]. The incidence of chronic burn pain is related to the levels and duration of acute pain [19].

Acute pain evoked by thermal burns causes stimulation of the skin nociceptors and is transmitted to the cerebral cortex through three pathways [20,21], while in the periphery neurotransmitter release, axon reflex, redness, swelling, tenderness and release of inflammatory response mediators occur. These phenomena increase the sensitivity of nociceptors, causing the so-called primary hyperalgesia [18,21,22]; furthermore, the continuous stimulation of nociceptive afferent fibers determines excitability of dorsal horn and hypersensitivity of surrounded undamaged skin, the so-called secondary hyperalgesia [18,21], that may cause chronic pain [22,23].

In burn patients experience pain depends on the quality of pain [18]. The background pain [20,24] is exacerbated by different types of pain, characteristically of high intensity and short duration, the procedural pain[20,24], the unpredictable breakthrough pain and the postoperative pain [18,20,24,25]. Tissue regeneration is associated with patient discomfort (tingling and itching sensation) [22]. After the wounds have healed, burn patients could experience “neuropathic pain”. The International Association for the Study of Pain defines it as “pain initiated or caused by a primary lesion or dysfunction in the nervous system” [26]. It has been described as burning, “stabbing, shooting, pinsand needles, and electric shock-like sensations” [27].

The most beneficial effects of DEX seem to be apparent in the perioperative phase, as documented by Scheinin et al, who described that DEX reduced the requirement of opioids perioperatively in the recovery room [28]; the analgesic requirements of burn patients are different compared to the general population due to higher sensitivity and adapting pain levels caused by the high frequency of surgical procedures or dressing changes. DEX showed a major contribution to patients sedation and well being in the setting of the burn ICU.

In a study designed to compare analgesic efficacy and side effects of oral dexmedetomidine and ketamine in adults for burn wound dressing, Kundra et al. [29] divided randomly into 2 groups sixty healthy adults with thermal burns: the first group received 5 mg/kg ketamine and the second group 4 mcg/kg oral DEX.

Even if the mean VAS score was significantly reduced from baseline in both the groups at all time points (P < 0.05), in the first group pain relief (overall mean VAS 2.6 ± 0.6 cm) was significantly better when compared to second group (overall mean VAS 3.8 ± 0.8 cm), the related patients were significantly more sedated (median 3) when compared to second group (median 2, P < 0.05) and the main complications observed were delirium and excessive salivation. Finally more patients preferred ketamine (63.3%) than dexmedetomidine (36.7%), P < 0.05.

Although the dex has sedative and analgesic properties, it is less effective than ketamine for acute situations management in burns ICU [29].

Ketamine is a rapid, safe and effective anaesthetic agent during burns debridement and dressing changes, with few contraindications. Propofol, used for its favourable pharmacokinetics, lacks the analgesic property intrinsic to ketamine, whereby fentanyl is added to compliment its analgesic property.

Given the pharmacological properties of these drugs, we can expect that concomitant dexmedetomidine use may reduce the requirement of propofol and ketamine, with faster postoperative recovery and more stable intraoperative haemodynamics.

For this purpose, sixty adult patients posted for elective debridement and dressing included in the study of Ravipati et al. [30] were divided into two groups: thirty patients received intramuscular DEX, 1 μg/kg, 1 h before shifting to the operation theatre, while the other thirty did not. A titrated infusion of propofol and ketamine followed to get a score of Ramsay Sedation Scale (RSS) six in all patients. Intraoperatively haemodynamic parameters were recorded at regular intervals of 5, 15, 30, 45, and 60 min.

At the end of the surgical procedure, the drug infusion was discontinued; the total drug consumption and the recovery time (i.e. the time from discontinuation of infusion of the study drug and achievement of RSS score of 3) was noted.

The mean doses of ketamine and propofol used in DEX group (100.5 ± 17.58 mg and 127.7 ± 15.47mg) were significantly less than in control group (231.5 ± 60.39 mg and 254 ± 59.22 mg respectively). Time to recovery was significantly lower in the control group (9.57 ± 1.50 min vs. 11.53 ± 2.56 min)

The choice of the dex dose used in the study comes from the evaluation that most previous investigations have proven the cardiovascular depressive effects of i.m. DEX at a dose of 2.5 μg/kg, which increases the incidence of hypotension and bradycardia, whilst Virkkila et al. [31] has shown that i.m. DEX 1 μg/kg produces sedation with minimal haemodynamic side effects when given as premedication.

Adding DEX with ketamine shows the great advantage of balancing haemodynamic and adverse effects of each other: DEX decreases the incidence of tachycardia, hypertension, salivation, and emergence phenomena from ketamine, while ketamine prevents bradycardia and hypotension of DEX. Furthermore ketamine speeds the onset time of sedation, tipically slow for i.m. DEX [30].

DEX (1 μg/kg i.m. dose) is a good anaesthetic adjuvant that decreases the requirement of propofol and ketamine during burns debridement and dressings, reduces sympathoadrenal response, provides hemodynamic stability and adequate levels of analgesia, and finally has an excellent recovery profile.

## IV. ROLE IN PEDIATRIC ICU

Dex has been used as an off-label drug for sedation of children in the intensive care unit [32,33]; the preliminary experience in burned pediatric patients did suggest opioid sparing effects [34].

Sedation in children can be even more difficult to achieve because of parental separation, stranger fear, incomprehension, and degree of perceived situational control.

Hsin Lin et al. [35] describe the dosing, safety, and efficacy of DEX for sustained sedation in intubated pediatric burn patients, treated between 2005 and 2008. Patients served as their own controls using the time periods when they received sedatives other than DEX.

Eleven patients with 17 DEX treatment courses were identified. The median patient age was 7 years (range 1.6–17 years), and median burn size was 30.5% TBSA (range 6–59%). Patients were ventilated for a median of 9 days (range 4–46 days). The median initial dose of DEX was 0.39 gamma/kg/hr (range 0.10–1.16/gamma/kg/hr), with a median infusion dose of 0.57 gamma/kg/hr (range 0.11–1.17 gamma/kg/hr) and median treatment duration of 40 hours (range 1–356 hours). None of the patients received DEX loading dose. Patients achieved more appropriate Riker scores while treated with DEX than while being treated with other sedatives (3.8 vs 3.3, *P* 0.003). The incidence of hypotension and/or bradycardia while on DEX was not greater than when it was not being used. Median length of hospital stay was 49 days (range 7–118 days).

DEX seemed to be safe and effective for sedation of pediatric burn patients on mechanical ventilation [35].

A prospective, randomized study of DEX vs midazolam in mechanically ventilated infants and children found that DEX at a dose of 0.25 mcg/kg/hr was equivalent to midazolam at 0.22 mg/kg/hr. DEX at 0.5 mcg/kg/hr was found to be superior to midazolam for sedation. It had fewer morphine bolus doses, decreased 24-hour morphine needs and decreased remarks of inadequate sedation [32].

The hemodynamic consequences and the sympatholytic effects [36–38] of DEX administration, at higher doses in pediatric burn patients, have been systemically studied with prospective preliminary study by examining the hemodynamic changes during bolus and continuous infusion of Dex administered in sequence [36].

Eight intubated patients with ≥20 to 79% TBSA received a 1.0 μg/kg bolus of Dex followed by an ascending dose infusion protocol (0.7–2.5 μg/kg/hr), with each dose administered for 15 minutes. Significant hypotension and decrease in heart rate were registered, but no bradycardia (HR < 60) or heart blocks were observed. In three patients the bolus dose of Dex decreased MAP to <50 mmHg; three patients received the highest infusion dose of Dex (2.5 μg/kg/hr), whereas in 2 patients the infusion was stopped due to persistent hypotension (MAP < 50 mm Hg). These data indicate that in critically injured pediatric burn patients a bolus dose of Dex (1.0 μg/kg for 10 minutes) and high infusion rates may require countermeasures (fluid resuscitation or vasopressor support) to maintain normotension [36].

## V. HAEMODYNAMIC CHANGES IN BURN PATIENTS

The hypotension observed in burned patients is a frequent phenomenon. The pathophysiology of burn injury involves a persistent circulating levels of epinephrine, norepinephrine, and dopamine [39,40] and an increased renin-angiotensin activity, responsible for inducing hypertension or maintening normotension even during relative hypovolemia [41–43]. In these patients, the sympatholysis and the MAP decrease produced by Dex can occur more easily.

The hypotensive response to sedative drugs, like morphine and midazolam, is well documented and mediated by central and peripheral mechanisms [44,45], also in burned patients. Indeed, a 24-hour resuscitation volume of 4 mg/kg percent burn is applied by the original Parkland formula.

Recognition of increased pain scores in posttraumatic stress disorders [46–48] and consequent liberal sedation policy of burn caregivers needed an increased intravenous fluid requirements [49,50].

Decreasing morphine and midazolam doses, or volume/pressor support *prior* to Dex initiation, might prevent drops in blood pressure [36]. Finally the opioid-sparing effects of Dex requires to assess the pretreatment volume status and to evaluate rescue measures to support the hemodynamic changes [36].

Walker et al. [12] conducted a retrospective chart review of 65 pediatric burn patients (42 boys, 23 girls) in the ICU who received DEX infusion because of failure to achieve adequate sedation with their standard regimen of opioids and benzodiazepines at Shriners Hospital for Children, Cincinnati.

The average duration of DEX was 11 days (range: 2–50), with a mean DEX dose of 0.5 mcg/kg/hr. With DEX titration, all patients were rated “adequately sedated,” even though all were sedation failures with opioids and benzodiazepines. Eleven of 42 patients receiving ventilatory support were extubated while on DEX infusion, and no patient showed evidence of DEX induced respiratory depression. Infusions were weaned over the course of treatment without evidence of rebound hypertension or withdrawal, indicating that the longterm use of DEX may be safe. No tachyphylaxis was noted[12].

## VI. ROLE OF DEX IN ACTUAL GUIDELINES

A very variable tolerance of sedative drugs in some patient populations needs to have multiple agents available for management of patients in ICU. This makes it difficult to apply the findings of randomized controlled trials in clinical practice and to evaluate real effectiveness of changes in this context [51].

Over the last years, it is a considerable evidence that both choice of a drug and way of its use can significantly change patient outcomes. This, in turn, has influenced pain, agitation and delirium guidelines [52] that suggest a shift in favor of non-benzodiazepine alternatives, as DEX or propofol.

The clinical effectiveness of a sedation protocol providing an early use of DEX in place of benzodiazepines was assessed in a “before-after” study. Patients required continuous sedation (midazolam, propofol or DEX) and mechanically ventilated for at least 24 hours in the surgical ICU (SICU) or medical ICU (MICU) and included in the first group modified the sedation protocol, minimizing use of benzodiazepine infusions and favoring early use of DEX [51].

An initial DEX infusion of 0.5 μg/kg/hr (range of 0.2 to 1.5 μg/kg/hr) without boluses was applied.. In both phases, bolus doses of midazolam were administered for the management of breakthrough agitation. Propofol and midazolam were also allowed in the after phase according to physician discretion. A fentanyl infusion and/or boluses as needed was the main treatment of pain in both phases.

This protocol, that reduces benzodiazepine use and targets light sedation, lead to significant improvements in the duration of mechanical ventilation and the requirement for tracheostomy. In addition, fentanyl infusion for pain, used in both group, was significantly reduced in the after phase, because of the reduced time on mechanical ventilation, the supplementary analgesic properties of DEX or an improved ability to assess pain in the after phase [51].

Significant hypotension or bradycardia were not observed between groups, and the percentage of patients requiring the addition of a vasopressor was lower in the after phase. DEX failure occurred in one in four patients, because hypotension [51].

A minimized sedation improves patient outcomes [53–58]. A sedation protocol targeting light sedation as standard of care using continuous infusions of DEX provides a significant reduction in time on the ventilator, but not a statistically significant reduction in ICU length of stay [51].

On December 21, 2015 Italian Drug Agency [59] authorized the insertion of drug dexmedetomidine (Dexdor) in the list of drugs distributed in the total load of the NHS for the following therapeutic indications: procedural analgo-sedation out of the operating room (Not Operating Room Anesthesia NORA) in children with difficult airway management and in children with seizure disorders that should be subjected to diagnostic studies to localize epileptic foci, and analgo-sedation in critical infants and children in ICU, mechanically ventilated and poorly responsive to conventional analgo-sedative treatment.

Inclusion criteria for algo-sedation of critical infants and children in ICU are defined:

- ICU admission
- mechanical ventilation
- continuous analgo-sedative treatment for at least five days
- persistent high values of the score evaluation of analgo-sedation despite attempts to increase the dosage of administered analgesics and sedatives
- Informed consent by the parents/legal guardians

Exclusion criteria are:

- known or suspected hypersensitivity to the drug
- severe bradycardia
- severe hypotension
- ongoing treatment of clonidine or other alpha agonist

Under the guidance of the German Society of Anaesthesiology and Intensive Care Medicine (DGAI) and German Interdisciplinary Association for Intensive Care and Emergency Medicine (DIVI), a new version of “evidence and consensus based guideline for the management of delirium, analgesia, and sedation in intensive care medicine” [60] were developed to provide practical guidance for the symptom-based prevention, diagnostics and therapy of delirium, anxiety, and agitation, as well as for the protocol-based analgesia, sedation, and sleep management during critical illness. An early management of these symptoms improves recovery and longterm outcome, while reducing post-intensive-care-unit syndrome (PICS) and mortality.

Aside from an adequate basic level of analgesia, additional analgesics (local and systemic) and/or procedural sedation may be necessary when performing various procedures (e.g. dressing changes). There are multimodal concepts for the use of analgesics, adjuvants, and non-pharmacological strategies regarding pain management in patients with severe burns [18]. Especially for analgesia and sedation of burn-injured children, the use of standardized protocols and training programs should be used [61].

For analgesia, the continuous intravenous use of lidocaine is not recommended [62], while the use of co-analgesics, such as gabapentin, may be considered adjunctively to opioids [63]. It is also suggested the use of ketamine to reduce secondary hyperalgesia [64,65] and the opioid demand [66] of burn patients and the use of α_2_-agonists for sedation, as they have been shown to be more effective on burn patients as other drugs (e.g. benzodiazepines) [1].

In burn-injured children, standardized protocols and training programs for analgesia and sedation during dressing changes are strongly recommended [61].

The combination of non-pharmacological procedures (massage in non-burned areas, hypnosis, and virtual reality) with opioids is more effective at alleviating pain than a single opioid analgesia [67–69].

For the of procedural pain, guidelines suggest the use of ketamine over opioids [70]. For procedural sedation during dressing changes in burned children, the use of dexmedetomidine may be considered [2].

These data recognize a concrete effectiveness of dexmedetomidine in the management of the burn patient, especially as a fundamental element for the control of pain, anxiety, delirium and to achieve an optimal level of sedation, correlated to potential improvement of the outcome.

## VII. DEX IN BURNS AND SEPSIS

Burns, such as other traumatic injuries, induce global changes to the systemic immune response, including suppressed immune function and increased susceptibility to infection. Moreover, remote organ injuries affecting kidney, lung, gut and bone marrow compartment are often associated with burn trauma in human and animal studies. This relationship between burn and remote organ injury supports the hypothesis that immune suppression may facilitate the translocation of gut-derived bacteria and/or their products and contribute to the development of systemic inflammatory response syndrome, sepsis, and multiple organ dysfunction syndrome in critically ill burn patients [71]

Progress over the last 50 years has led to a decline in mortality from ≈70% to ≈20% in the best series of patients with septic shock. In these cases, the mortality appears related to multiple organ failure linked to comorbidities and/or an intense inflammatory response: shortening the period that the subject is exposed to circulatory instability may further lower mortality. Treatment aims at reestablishing circulation within a “central” compartment (i.e., brain, heart, and lung) but fails to reestablish a disorganized microcirculation or an adequate response to noradrenaline, the most widely used vasopressor [72].

These evidence indicates a patchy and disperse maldistribution of O_2_ during sepsis, as opposed to an inability to utilize O_2_ [73], that is, a cytopathic hypoxia[74]. Increasing the delivery of oxygen to supranormal levels may not improve tissue oxygenation if the increased O_2_ supply cannot be properly distributed and early treatment aimed at restoring uniform distribution of O_2_ may lead to improve outcomes [73].

In two cases [75], the treatment with the α_2_-adrenoceptor agonist, clonidine (1 μg·kg^−1^·h^−1^), in addition to state-of-the-art treatment, reduced NA requirements in a patient presenting with HIV and terminal pulmonary sepsis [75] and a neonate presenting with necrotizing enterocolitis. In addition, this reduction in requirement for NA in rat [76] and sheep [77] experimental models of sepsis has been documented, using high and low doses, respectively, of the α_2_-adrenoceptor agonists, clonidine and DEX. Furthermore, the pressor responsiveness to a noncatecholaminergic vasopressor, angiotensin II, was also reduced by clonidine treatment [77].

One possible mechanism [78] for this effect of α_2_-adrenoceptor agonists in sepsis is that, during septic shock, as during exercise [79], there is increased sympathetic nerve activity and endogenous plasma catecholamines [80–82] with a downregulation in responsiveness to stimulation of α_1_- and β-adrenoceptors, which may result from reduced binding or reduced sensitivity/intracellular coupling. Conversely, after lowering plasma catecholamine concentrations with pharmacologically evoked α_2_-adrenoceptor agonists, as during rest after exercise, the downregulation of α_1_-adrenoceptors is converted to upregulation, with an increased pressor response to vasopressors.

The evidence of the anti-inflammatory effect of DEX was obtained in several experimental animal models. The drug administration in models of induced sepsis, inflammation, ischemia-reperfusion injury and trauma showed its protective effect, with an overall reduction of the inflammatory response, proven by the finding of reduced levels of the major mediators of systemic inflammation: IL-6 and TNF-α mRNA and respective proteins, mRNA for TLR4 and MyD88, NFkB mRNA, IFN-γ and IL-4 mRNA, protein HMGB1 [83–90].

Similar results were obtained in different cell models in which the inflammatory response induced by exposure to LPS was significantly reduced after DEX incubation[91–94].

Finally, the drug effectiveness to modulate the main inflammatory mediators levels has been confirmed in several clinical trials that investigated the administration of dex in patients undergoing surgical procedures (hepatectomy, laparoscopic cholecystectomy, abdominal surgery, oncologic surgery, cardiopulmonary bypass) or in ICU septic patients [95–98].

These clinical trials suggest that α_2_-adrenoceptor agonists lead to a sympathetic deactivation with a reversal of the peripheral microcirculatory shut-down, and reduce inflammation and multiple organ failure, proposing an effect of DEX on outcomes, including mortality, in sepsis.

However, evidence-based documentation of the effects of α_2-_agonists is needed in the setting of human septic shock, and an end-point on mortality would require a large sample, not compatible with these preliminary trials.
